# Measuring the burden of arboviral diseases: the spectrum of morbidity and mortality from four prevalent infections

**DOI:** 10.1186/1478-7954-9-1

**Published:** 2011-01-10

**Authors:** A Desirée LaBeaud, Fatima Bashir, Charles H King

**Affiliations:** 1 Center for Immunobiology and Vaccine Development, Children's Hospital Oakland Research Institute, Oakland, California, USA; 2 Center for Global Health and Diseases, Case Western Reserve University, Cleveland, Ohio, USA

## Abstract

**Background:**

Globally, arthropod-borne virus infections are increasingly common causes of severe febrile disease that can progress to long-term physical or cognitive impairment or result in early death. Because of the large populations at risk, it has been suggested that these outcomes represent a substantial health deficit not captured by current global disease burden assessments.

**Methods:**

We reviewed newly available data on disease incidence and outcomes to critically evaluate the disease burden (as measured by disability-adjusted life years, or DALYs) caused by yellow fever virus (YFV), Japanese encephalitis virus (JEV), chikungunya virus (CHIKV), and Rift Valley fever virus (RVFV). We searched available literature and official reports on these viruses combined with the terms "outbreak(s)," "complication(s)," "disability," "quality of life," "DALY," and "QALY," focusing on reports since 2000. We screened 210 published studies, with 38 selected for inclusion. Data on average incidence, duration, age at onset, mortality, and severity of acute and chronic outcomes were used to create DALY estimates for 2005, using the approach of the current Global Burden of Disease framework.

**Results:**

Given the limitations of available data, nondiscounted, unweighted DALYs attributable to YFV, JEV, CHIKV, and RVFV were estimated to fall between 300,000 and 5,000,000 for 2005. YFV was the most prevalent infection of the four viruses evaluated, although a higher proportion of the world's population lives in countries at risk for CHIKV and JEV. Early mortality and long-term, related chronic conditions provided the largest DALY components for each disease. The better known, short-term viral febrile syndromes caused by these viruses contributed relatively lower proportions of the overall DALY scores.

**Conclusions:**

Limitations in health systems in endemic areas undoubtedly lead to underestimation of arbovirus incidence and related complications. However, improving diagnostics and better understanding of the late secondary results of infection now give a first approximation of the current disease burden from these widespread serious infections. Arbovirus control and prevention remains a high priority, both because of the current disease burden and the significant threat of the re-emergence of these viruses among much larger groups of susceptible populations.

## Background

Arthropod-borne viral infections, or arboviral infections, are common causes of disabling fever syndromes worldwide, but their cumulative impact on global disease burden has not been fully assessed. In their acute stages, arboviral infections cause a broad spectrum of disease, ranging from asymptomatic infection to severe undifferentiated fever. They can also progress to much more complex secondary conditions, or sequelae, such as encephalitis or hemorrhagic diathesis, which result in long-term physical and cognitive impairment or in early death [1,2].

**Figure 1:**
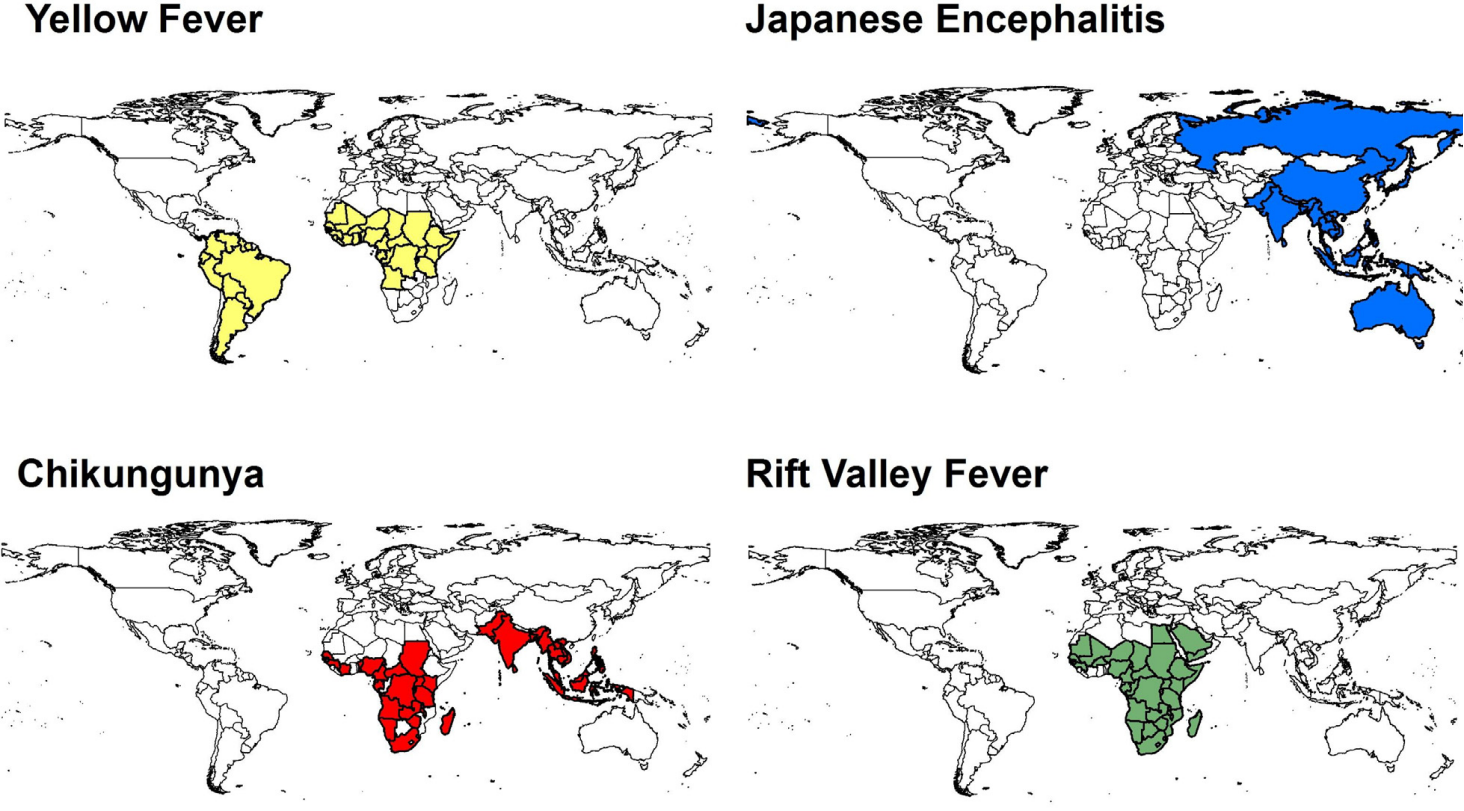
**Countries having transmission of the four arthropod-borne viruses included in this study: yellow fever virus, Japanese encephalitis virus, chikungunya virus, and Rift Valley fever virus**.

More than 100 arboviruses are known to cause disease in humans. A significant subset, including members of the *Flaviviridae*, *Bunyaviridae*, and *Togaviridae *families, are transmitted widely in different areas of the world (Figure 1). As threats to public health, these viruses are best known for their propensity to cause encephalitis and/or viral hemorrhagic fever (VHF) syndromes [1-4]. Arboviruses are also considered to be emerging pathogens based on their geographic spread and their increasing impact on susceptible human populations [5-14]. As an example, dengue virus (DENV) infections, once rare, are now estimated to cause > 50 million clinical cases per year following a resurgence in Asia and renewed spread through Central and South America [15].

As with other tropical diseases, the disabling sequelae of arboviruses disproportionately affect resource-poor communities through chronic impairment of individual performance in activities of daily living [16]. Long-term longitudinal studies of the impact of childhood arbovirus infections remain few in number, particularly for post-encephalitic complications, meaning that the lifetime burden of these infections is not well understood [17-19]. Of note, it is not just the severe forms of acute arbovirus-related illness but also the nominally milder forms of arboviral infection that can result in long-lasting impairment, as has been best described for neurologic and ophthalmologic complications of West Nile virus (WNV) infection [20-26]. Frequent neurological impairment, lasting weeks to years, has now also been recognized in association with chikungunya virus (CHIKV) and dengue infection [2]. Less well known are the medium- to long-term (months to years duration) ocular complications that can occur after arboviral infection. Rift Valley fever virus (RVFV) [27-32], WNV [33-39], DENV [40-47], and CHIKV [48-51] all cause inflammatory ophthalmologic complications, including chorioretinitis, uveitis, iridocyclitis, and optic neuritis. With WNV, when such post-infectious, long-term sequelae occur, most patients recover within the two years following the onset of illness [52-54]. However, this is not the case for Japanese encephalitis virus (JEV) infection, in which post-encephalitic neurologic injury and disability are most often permanent [55,56]. Newer evidence also suggests that CHIKV infection can also cause significant physical and mental impairment for > 30 months after infection [57]. At present, only the disease burden caused by dengue and JEV infections are included in World Health Organization (WHO) Global Burden of Disease (GBD) estimates [58]. For other arboviruses, any significant cause-specific early mortality or any long-term infection-related morbidity (particularly as a lifetime effect of childhood disease) represent a substantial health deficit not currently captured by disease burden assessments, and hence are not included in top-level discussions of disease control priorities [58-61].

As new evidence has emerged in the past decade on the frequency of arboviral infections and the incidence of their long-term, disabling sequelae [17-37,39-54,62], it is now appropriate to revisit the global estimates of the health impact of these infections. In this paper, our aim is to review the available evidence on incidence and disease duration and critically compare the estimated disease burden measured in disability-adjusted life years (DALYs) caused by four common arboviral infections:

1) Yellow fever (YFV), a disease with very high acute mortality due to hemorrhagic complications [63];

2) Japanese encephalitis (JEV), having high rates of both acute mortality and very long-term neurologic disability [55,56,64];

3) Chikungunya virus (CHIKV) fever, a disease with low mortality but high rates of post-infectious rheumatologic and neurologic morbidity [48,65,66];

4) Rift Valley fever (RVFV), having intermediate rates of mortality and of long-term ocular and neurologic morbidity [28,30,31,67,68].

## Methods

### Setting for the analysis

In deriving our disease burden assessments for the arboviruses under study, we chose to develop DALY estimates for the disease comparisons presented in this paper. To do so, we incorporated the following assumptions:

• The population of interest is the 2005 world population, listed by country, as quantified according to the estimates given in the United States Census Bureau's International Database (http://www.census.gov/ipc/www/idb/index.php).

• The global and regional disease burden of each study disease is measurable in DALYs, developed as previously described in WHO and World Bank publications [59] and modified by the Disease Control Priorities Project [61].

• DALY scores for each condition represent the sum of two components: a) for cases of mortality, the years of healthy life lost (YLL) from a standard expected years of life lost (SEYLL), plus b) for individuals having nonlethal, disease-specific disability, the years lived with disability times a disability weight reflecting the proportion of impairment caused by that health condition (YLD) [69].

• Different from the original DALY concept [69], which employed age-weighting, and in accord with more recent usage [60], we report both 3% time-discounted, unweighted DALYs (indicated as DALY (3,0)) and nondiscounted, unweighted DALYs (indicated as DALY (0,0)) for each condition. [See [69] for a detailed discussion on age-weighting and discounting.] Our intention is to make the current estimates align with contemporary discussions of global health risks and the Global Burden of Disease (http://www.who.int/healthinfo/global_burden_disease/en/index.html).

### Data collection

DALY summary calculations rely on the best available, evidence-based estimates of yearly incidence, mortality, average age at death, and, for nonlethal cases, information on the duration and impact (disability weight) of cause-specific disability outcomes related to the disease. We searched the available published literature and official reports on YFV, JEV, CHIKV and RVFV, focusing on the years since 2000. In order to capture the impact of endemic disease, we excluded studies and reports of disease among travelers from nonendemic areas. Searching was initiated with the use of the arbovirus name and the terms "outbreaks," "complications," "disability," "quality of life," "DALY," and "QALY" in PubMed and Google Scholar, and continued with hand searching of bibliographies of the selected publications. Each publication or report was evaluated by two authors, and the relevant information was extracted. Publications had to meet three inclusion criteria: 1) discussion of complications that lead to mortality or prolonged morbidity; 2) focus on population-based information, including incidence and/or prevalence; and 3) published after 2000. We screened 210 published studies, of which 38 were selected for inclusion. Additional tables and charts from US Centers for Disease Control and Prevention and WHO publications were included in the data collection.

### DALY calculations

Nondiscounted and time-discounted DALY estimates for each disease were calculated according to standard methods (but with only uniform age weighting [69]) using spreadsheets developed by the authors. Because of limitations in the available data (see Results), our summary estimates were calculated for both sexes together, and not distributed according to age group, as ideally presented in DALY tables [59,60]. Additionally, we present our DALY estimates as a range of credible values based on uncertainties or potential variability of the input values. The range of inputs for incidence of symptomatic cases, associated case fatality, median age at death, and duration of post-infection disability were taken from the included reports.

Disability weights (DW) for acute JEV disease and chronic sequelae were taken from Murray and Lopez [59] and Mathers et al. [58] (Table 1). For the other study arboviruses, the range of DWs for severe acute febrile illness were based on published estimates for analogous acute viral syndromes that were ranked in the original GBD framework, i.e., dengue (DW = 0.172 - 0.211) or JEV (DW = 0.616) [59]. DWs for chronic neurological and visual impairment sequelae of the studied arbovirus infections were based on closely analogous syndromes ranked within the GBD framework, i.e., those for post-encephalitic cognitive impairment (DW = 0.469-0.485) and other neurologic sequelae (DW = 0.388-0.468) listed for JEV, for lameness after poliomyelitis (DW = 0.369), and for infection-related blindness (DW = 0.600) or low vision (0.223-0.245) following trachoma [59] (see Table 1).

**Table 1:** Sources for disability weight estimates of arbovirus-related long-term morbidities: Proxy values based on Global Burden of Disease project disability weights for analogous health states

Arbovirus	Sequelae details and expected prevalence	**Analogous morbidity DW from GBD project listings **[59]	DW input range for YLD estimations
Yellow fever (YFV)	Zero to 2% of YFV survivors who require critical care for severe hemorrhage or acute encephalopathy can expect protracted symptoms ranging from mild cognitive impairment to severe disability [79]	Persisting mild cognitive impairment ≈ malnutrition, DW = 0.024 Severe psychomotor deficits ≈ severe complications of JEV, DW = 0.616	0.02-0.62

Japanese encephalitis (JEV)	30% to 50% of survivors can expect severe neurologic disability [72,83]	JEV-associated moderate cognitive impairment up to severe disability [59]	0.39-0.49

Chikungunya (CHIKV)	5% to 50% of survivors will have prolonged post-infectious rheumatologic and neurologic complications [57,85,86]	≈ Osteoarthritis, DW = 0.156 or ≈ Rheumatoid arthritis, DW = 0.233	0.16-0.23

Rift Valley fever (RVFV)	4% to 10% of survivors develop prolonged ocular and neurologic complications of ophthalmitis and meningoencephalitis [27,67]	Prolonged visual impairment ≈ trachoma, DW = 0.223 Severe psychomotor deficits ≈ severe complications of JEV, DW = 0.616	0.22-0.62

Abbreviations: GBD, Global Burden of Diseases; DW, disability weight; YLD, Years lived with disability

### Note on incidence estimates for arbovirus infections

**Figure 2:**
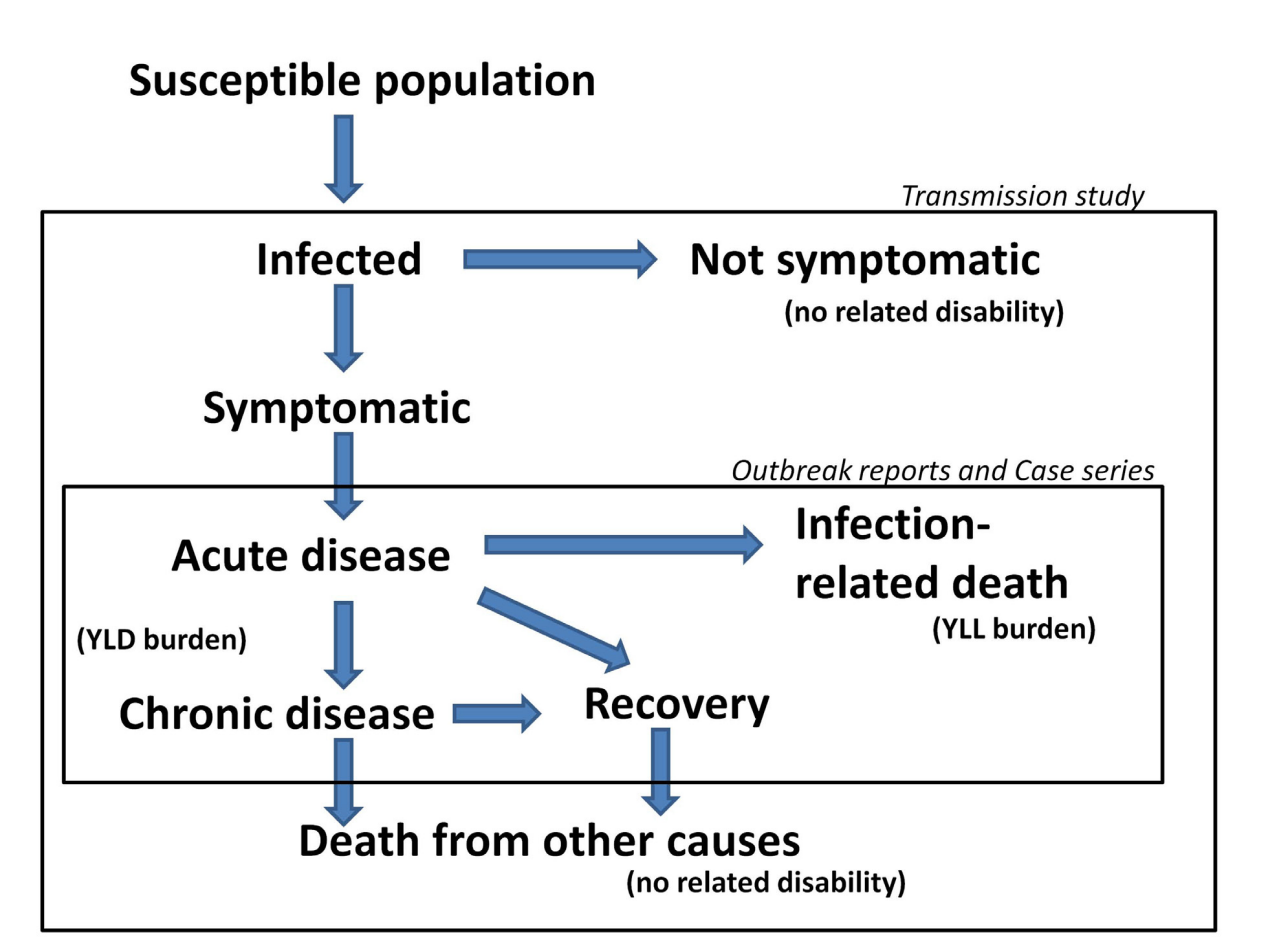
**Schema of disease development and assessment of population-level disease parameters for arboviral infections**. Among a general population, susceptible (nonimmune) persons who are exposed to the bite of infected/infectious arthropods will go on to develop infection, which may become symptomatic or remain nonsymptomatic. Transmission studies, usually based on serologic testing, provide evidence of past and present infections among the target population, but do not quantify human disease or disability. Symptomatic disease (acute and chronic) and cause-specific fatality may be tallied either actively (by public health outbreak investigations) or passively (by health care systems statistics or case series reports). The generalizability of the rates reported for complications (transition to chronic disease) and for arbovirus-related fatality can vary widely depending on the sampling frame, study design, duration of longitudinal follow-up, and accuracy of diagnostics used.

Arbovirus infections typically occur in epidemics. Due to seasonal and weather-related changes in arthropod-borne transmission of arboviruses and periodic fluctuations in the number of susceptible humans within an area [70,71], there may be significant variation in the local, regional, and global number of cases from year to year. Nevertheless, for planning purposes and for creation of comparable DALY estimates, it is important to estimate an annualized number of affected cases, as presented in the Results. It is also recognized that, for some arboviruses, only a small fraction of infected humans will become clinically symptomatic [72,73]. For this reason, our annualized incidence rates, case-fatality rates, and acute-to-chronic-disease case conversion rates are based on reported or informed estimates of the numbers of clinical cases of arbovirus-related symptomatic disease, and not on serologic evidence of local arbovirus transmission to humans (see schema in Figure 2). Although during epidemic periods, clinical case rates may reach 10-150 per 100,000 in a national population [1,71,74,75], over a period of one to three decades, annualized human disease incidence rates for symptomatic cases from the study arboviruses typically occur in the range of 0.2 per year per 100,000 general population (RVFV in East Africa [27,76]), to 2.4 per year per 100,000 general population (for JEV [77]).

Final DALY calculations based on 2005 estimates were as follows:

DALY = YLL + YLD_acute _+ YLD_chronic_, where

YLL = (Incident deaths) × (standard expected years of life lost at median age of death)

YLD_acute _= (Incident cases *with acute disease only*) × DW_acute _× (duration acute disease)

YLD_chronic _= (Incident cases progressing to chronic disease) × DW_chronic _× (duration of chronic disease). For calculation of DALY (3, 0) estimates, 3% per annum time-discounting was applied to future SEYLL and disease duration values.

### Sensitivity analysis

Because of substantial uncertainty about a number of parameters involved in the DALY calculation for each study arbovirus, we were unable to provide a "base case" for subsequent sensitivity analysis. Instead, we present what we believe is the most credible range for possible DALY burdens for each virus and discuss the implications for health planning assessments.

## Results

### Global distribution of disease

Table 2 and Figure 1 summarize the global distribution of the four study arboviruses. Included are estimates of the populations living in affected countries, along with estimates of annualized average cause-specific mortality rates and incidence of chronic disease cases due to the four arbovirus infections.

**Table 2:** World population affected by study arboviruses

Arbovirus	Number of affected countries	Population in endemic countries (% of global population)	**Estimated deaths per year**^**a**^	**Chronic cases per year**^**a**^
Yellow fever	44	972,155,287 (15%)	675-30,000	0 to 1,955

Japanese encephalitis	23	3,706,910,037 (57%)	3,500 to 15,000	7,350 to 22,500

Chikungunya	41	2,548,842,950 (39%)	33 to 25,761	1,193 to 46,453

Rift Valley fever	32	778,528,381 (12%)	4 to 91	12 to 272

^a ^Note: These are annualized averages over combined epidemic and interepidemic periods.

Table 3 summarizes information abstracted by our literature review regarding the estimated average number of incident clinical cases of arbovirus infection-related disease each year, along with the median age of onset for symptomatic disease, the range or reported case-fatality rates (per symptomatic case), and estimates of the risk for progression to multiyear or permanent disability following the onset of symptomatic illness due to infection. Evidence from the available literature suggests that YFV may be the most prevalent infection of the four viruses evaluated, in part because of declines in YFV control efforts over the last several decades [78,79], and in part due to the success of JEV vaccination programs that have reduced the annual number of worldwide JEV cases to 50,000 or fewer [72].

**Table 3:** DALY inputs: Average annualized incidence, typical age at onset, mortality, and risk for disability for studied pathogens

Arbovirus name (common abbreviation)	**Estimated clinical cases**^**a **^**per annum**^**b**^	Median age for symptomatic disease	**Case-fatality rate (%)**^**c**^	Survivor's risk for multiyear or permanent disability
Yellow fever (YFV)	30,000 to 200,000 [78]	25 yr [80,81]	2 to 15% [78,79,81]	0 to 2% [78]

Japanese encephalitis (JEV)	35,000 to 50,000 [72,82]	10 yr [72]	10 to 30% [72,82]	30 to 50% [72,83]

Chikungunya (CHIKV)	33,000 to 93,000^b^	40 yr [84,88]	0.1 to 28% [75,84]	5 to 50% [85,86]

Rift Valley fever (RVFV)	350 to 2,750^b^	28 yr [68,104]	1 to 3.3% [32,74]	4 to 10% [27,31]

^a ^Clinical cases are defined as symptomatic cases requiring medical attention. Asymptomatic or inapparent infections are not included in these figures.

^b ^Arbovirus transmission typically occurs in epidemic waves based on cyclical weather phenomena that contribute to significant increases in local abundance of arthropod vectors. For standardized DALY comparison, where global estimates of case rates were not available, the study assumed an annualized arboviral clinical case rate of 1.3 to 3.7/100,000 in the general population of an at-risk country.

^c ^Rate per clinical cases (see note a, above)

To summarize specific findings of the literature review regarding the individual study viruses:

YFV is associated with high acute mortality related to its recognized potential to progress to its toxic phase and hemorrhagic fever in 15% to 25% of early symptomatic cases [79]. This advanced form of disease has 20% to 50% mortality [78-80]. During outbreaks, the median age of patients is between 20 and 30 years old [1,81], and the duration of acute illness is typically 18 to 21 days overall. Symptoms of infection are high fever, chills, headache, muscle aches, vomiting, and backache, indicating an acute disability in the range of that for dengue hemorrhagic fever or acute JEV infection (DW = 0.2 to 0.6). Because the toxic phase of infection can lead to shock, bleeding, kidney and liver failure, and the need for several weeks of critical care, we also postulate a possible 10% incidence of long-term disabling complications after successful recovery from this critical form of the disease. In other words, about 1% to 2% of all nonlethal symptomatic YFV infections are estimated to result in chronic disabling disease.

JEV has a high acute mortality rate (10% to 30%) [72,82] and for survivors, it frequently causes long-lasting morbidity due to complex neurological complications [72,83]. The median age of patients is less than 10 years old among unvaccinated populations, with the majority of deaths occurring in this phase of childhood [83]. The duration of acute, nonlethal disease (DW = 0.616) is two to three weeks. Although some motor deficits and movement disorders improve after the acute illness, 30% to 50% of JEV survivors have long-term neurologic or psychiatric sequelae [72,83]. These include seizures, upper and lower motor neuron weakness, cerebellar and extrapyramidal signs, flexion deformities of the arms, hyperextension of the legs, cognitive deficits, language impairment, learning difficulties, and behavioral problems. These late outcomes of JEV infection are probably permanent and have been rated at DW = 0.388 to 0.485 by the Global Burden of Disease project [59,60].

CHIKV infection has lower mortality among the general population (1 per 1,000) [75], but higher mortality (14% to 30%) among hospitalized cases with neurological complications [84]. Symptomatic acute infection is associated with neurological, renal, cardiac, respiratory, hepatic, and hematological complications, with a high risk for long term (> 30 mos) rheumatologic complications among patients older than 30 years [57,85,86]. Those patients affected by long-term, chronic symptoms after CHIKV infection reported significant persistent pain and impairment of activities of daily living [57,87], with resulting decreased quality of life as measured by formal testing [57,85]. For this post-chikungunya syndrome, we and others [88] have chosen to use DW values of 0.11 to 0.23 as analogous to values for chronic arthritides in the Global Burden of Disease framework [59,60].

RVFV is associated with a wide range of morbidities. Human RVFV infection is considered to be nearly always symptomatic [89,90], typically presenting as a syndrome of fever with nausea and arthralgias [68], sometimes progressing to meningoencephalitis (10%) [32,67,68], uveitis/retinitis (10% to 30%) [27,31], or hemorrhagic diathesis (1% to 3%) that is highly fatal [32,67,68]. Average age of symptomatic disease is between 20 and 30 years [68,76]. Average duration of acute RVFV infection is one to two weeks, while late neurological and visual complications are most likely lifelong [27,67]. Disability from these late complications was considered to be analogous to the severe neurological sequelae of JEV or its cognitive impairment (DW = 0.39 and 0.47, respectively), and to low vision (DW = 0.22) or blindness (DW = 0.60) from trachoma.

Table 4 indicates the range of calculated global 2005 DALY estimates for each study virus based on the information summarized above. Because of variability in estimates for the incidence of the study arboviruses and the conditional probability of death or chronic complications after acute disease, we calculated a range of DALY values for each pathogen based on an annualized average number of symptomatic cases each year. Our results indicate a probable range for the aggregate, nondiscounted, uniformly weighted DALY (0,0) burden equal to 307,082 to 5,057,081 DALYs, and a 3% discounted, uniformly weighted DALY (3,0) of 127,681 to 2,385,203 for these pathogens combined for the world population.

**Table 4:** Calculated global 2005 DALY estimates for the four study viruses--yellow fever, Japanese encephalitis, chikungunya, and Rift Valley fever, based on data from previous tables

2005 Nondiscounted DALYs (0,0)						
**Arbovirus**	**2005 YLLs**	**2005 YLDs**	**2005 DALYs (0,0)^a^**	**YLLs per death**	**YLDs per acute case**	**YLDs per chronic case**

Yellow fever	38,475-1,710,000	303-73,704	38,827-1,774,049	57	0.012-0.024	1.43-35.3

Japanese encephalitis	252,000-1,080,000	13,660-1,002,006	265,778-1,859,170	72	0.012-0.024	1.43-44.5

Chikungunya	1,386-1,081,962	405-456,898	2,124-1,411,904	2-42	0.012-0.024	0.22-9.79

Rift Valley fever	192-4,901	158-7,236	353-11,958	42	0.006-0.06	12.6-26

Total	292,053-3,876,863	14,526-1,539,844	307,082-5,057,081	--	--	--

**2005 Discounted DALYs (3,0)**						

**Arbovirus**	**2005 YLLs**	**2005 YLDs**	**2005 DALYs (3,0)^a^**	**YLLs per death**	**YLDs per acute case**	**YLDs per chronic case**

Yellow fever	18,225-810,000	303-37,732	18,577-842,769	27	0.012-0.024	0.55-16.9

Japanese encephalitis	101,500-435,000	4,557-412,506	107,435-755,670	29	0.012-0.024	0.6-18.3

Chikungunya	759-592,503	394-260,399	1,481-780,234	23	0.012-0.024	0.21-5.56

Rift Valley fever	96-2,450	90-4,186	188-6,530	27	0.006-0.06	7.16-14.8

Total	120,580-1,839,953	5,344-714,823	127,681-2,385,203	--	--	--

^a ^NOTE: Total DALY min and max do not sum across the YLL and YLD rows above because gains in one DALY category column (e.g., YLL) are necessarily offset by reductions in survival and reduction in possible YLDs.

## Discussion

The four arboviral infections were chosen to highlight the broad range of their associated mortality and short- and long-term disability. The estimates presented here extend earlier work on the DALY impact of arboviral infections on global health [60]. Our combined 2005 DALY (3,0) estimates of 127,681 to 2,385,203, based on disease and mortality rates taken from published research and official reports, are in line with previously published estimates for dengue (529,000 DALYs (3,0)) and for JEV (604,000 DALYs (3,0)) for 2001. The variation between estimates indicates the difficulty of deriving precise point estimates for these diseases based on currently available data [58]. Data were limited for age- and sex-specific case rates and for the likelihood of chronic sequelae from these four arboviral infections. Often, data were collected only during outbreaks, and therefore, interepidemic transmission of these infections was not included. Despite these limitations, the findings suggest a persistent, nonnegligible world disease burden due to arboviral disease that requires further attention in research and public health agendas.

Perceptions about the actual frequency of arboviral disease are changing. As better diagnostics are introduced in at-risk areas, more accurate case finding allows for better definition of the risk of symptomatic disease and the long-term sequelae of infection [91,92]. Inherent in the DALY calculations, it is YLL from cause-specific mortality and the years lived with long-term, advanced disability that provide the largest proportion of DALY values associated with these conditions. Careful, multiyear longitudinal follow-up (as reported for WNV in the US [20-22,24,25,93]) will be needed to define these rates more accurately.

Misclassification in diagnosis has also played a role in the underestimation of viral disease burden. Arbovirus infections are common causes of severe disease but mimic other infections, such as acute malaria, and are frequently misdiagnosed as such [4,94]. Better diagnosis and careful post-mortem studies indicate that a significant minority (23%) of WHO-defined "cerebral malaria" cases can be due to other infectious and noninfectious causes [95]. As true malaria prevalence wanes with successful prevention and treatment campaigns, arboviruses are poised to become the most frequent cause of severe febrile illness throughout the world. As shown in Figure 1 and Table 2, many countries and a large segment of the world's population are at risk for arboviral transmission. Of special note, malaria control programs using insecticide-treated bed nets and indoor residual spraying are not a panacea for arbovirus control. Though the night-biting Anopheles vectors of malaria are strongly affected by household-based interventions, many arboviruses are transmitted by daytime-feeding Aedes mosquitoes or by outdoor Culex spp. mosquitoes. Thus, arbovirus transmission often occurs outside the home while at work or in the market [79,96], meaning that very different interventions are needed for arbovirus vector control.

Arboviruses also have very important impacts outside the sphere of human health. An aspect of public health that cannot be assessed in the DALY system is the epidemic potential of most arboviral pathogens. While the DALY estimates reflect a "steady-state" assessment of disease burden in a given era, the highly unpredictable demands of arbovirus epidemics compel the need for policy planning regarding the provision of health care surge capacity, the prevention of economic and political disruption, and the blunting of the loss of food production due to zoonotic disease in arbovirus prevention and control. Although the current estimated clinical cases (Table 3) and DALY estimates (Table 4) may seem small when averaged per year over the world's population, substantial economic losses and health care disruption often result from a severe arboviral outbreak in a given region [97]. For example, once RVFV is known to be circulating in an animal herd, the World Organisation for Animal Health (OIE) places a three-year export embargo on those animals. The embargo is economically devastating to that farm and region, already suffering from the health consequences of the RVFV outbreak. The political, psychological, and economic implications of reporting arbovirus outbreaks may also contribute to intentional underreporting of these diseases.

As the world becomes increasingly networked through globalization and as vectors and viruses continue to evolve, infections that were once contained in remote tropical locations are likely to spread to new areas. Just as WNV emerged in the United States in 1999, other arboviral pathogens may escape control to infect large susceptible populations [7,98-101]. Recent examples include WNV [14] and chikungunya [102,103]. Because no specific medical treatment exists for most arboviral infections, continuing public health surveillance for vector-borne infections and continuing vector control (outside the standard health care delivery systems) are crucial to prevent these diseases and the morbidity and mortality that result from them. Likewise, vaccine development for arboviruses remains a priority because of their outbreak potential. Recent evidence suggests a surge in YFV cases due to the decline in YFV control and vaccination efforts, highlighting the precarious dominance we have over these infections and the immediate increase in disease burden resulting from lack of persistent focus on surveillance and control [78,79]. Ultimately, due to the externalities of arbovirus outbreaks, the development of new arboviral vaccines for common pathogens, such as dengue, may prove extremely cost-effective in terms of economic costs, even if their DALY impact is more modest.

## List of abbreviations

CDC: U.S. Centers for Disease Control and Prevention; CHIKV: Chikungunya virus; DALY: Disability-adjusted life year; DENV: Dengue virus; DW: Disability weight; JEV: Japanese encephalitis virus; RVFV: Rift Valley fever virus; SEYLL: Standard expected years of life lost; VHF: Viral hemorrhagic fever syndrome; WHO: World Health Association; WNV: West Nile virus; YFV: Yellow fever virus; YLL: Years of life lost; YLD: Years lived with disability

## Competing interests

The authors declare that they have no competing interests.

## Authors' contributions

Authors ADL and CHK were involved in the conception and design of the project, the acquisition of data, and their analysis and interpretation. Both were involved in drafting the manuscript and revision for content. Author FB had substantial participation in the acquisition and archiving of data and was involved in drafting the manuscript. All authors have given approval of the final version of the paper.
